# Complementary and Alternative Medicine for Long COVID: Scoping Review and Bibliometric Analysis

**DOI:** 10.1155/2022/7303393

**Published:** 2022-08-04

**Authors:** Tae-Hun Kim, Sae-Rom Jeon, Jung Won Kang, Sunoh Kwon

**Affiliations:** ^1^Korean Medicine Clinical Trial Center, Korean Medicine Hospital, Kyung Hee University, 23 Kyungheedae-ro, Dongdaemun-gu, Seoul 02447, Republic of Korea; ^2^Department of Acupuncture & Moxibustion, College of Korean Medicine, Kyung Hee University, 23 Kyungheedae-ro, Dongdaemun-gu, Seoul 02447, Republic of Korea; ^3^Korean Medicine Convergence Research Division, Korea Institute of Oriental Medicine, Daejeon 34054, Republic of Korea

## Abstract

Prolonged symptoms after the clearance of acute coronavirus disease 2019 (COVID-19) infection, termed long COVID, are an emerging threat to the post-COVID-19 era. Complementary and alternative medicine (CAM) interventions may play a significant role in the management of long COVID. The present study aimed to identify published studies on the use of CAM interventions for long COVID and provide an overview of the research status using bibliometric analysis. The present scoping review searched MEDLINE, Embase, and Cochrane Library from inception until November 2021 and identified published studies on CAM interventions for long COVID. A narrative analysis of the study types and effectiveness and safety of the CAM interventions are presented and a bibliometric analysis of citation information and references of the included publications were analyzed using the Bibliometrix package for R. An electronic database search identified 16 publications (2 clinical studies and 14 study protocols of systematic reviews or clinical studies) that were included in the present study. Dyspnea or pulmonary dysfunction, quality of life, olfactory dysfunction, and psychological symptoms after COVID-19 infection were assessed in the included publications. The two clinical studies suggested that Chinese herbal medications were effective in relieving symptoms of pulmonary dysfunction. Bibliometric analysis revealed the current trend of research publication in this area was driven by study protocols written by Chinese, Korean, and Indian authors. Thus, the present scoping review and bibliometric analysis revealed that there are few studies published about the use of CAM for long COVID and long-term management for COVID-19 survivors. Original studies on CAM interventions, including randomized controlled trials and systematic reviews, are required to actively support evidence for their use in the management of long COVID. PROSPERO registration: this trial is registered with CRD42021281526.

## 1. Introduction

Since the first report of the coronavirus disease 2019 case (COVID-19) in 2019, the impact of the pandemic has continued for more than 2 years worldwide. Although the number of people vaccinated has greatly increased, the number of new COVID-19 cases is still increasing due to factors, such as the delta and omicron variants [1]. However, a new health problem that is different from the acute increase of new confirmed cases is emerging, namely long COVID, which is defined as prolonged symptoms after the clearance of acute COVID-19 infection [2]. The number of patients experiencing long COVID is significant and is expected to increase as the COVID-19 pandemic continues. It is important to prepare for the post-COVID-19 era, and the healthcare strategy for long COVID patients is critical.

Several organs and systems are speculated to be involved in long COVID, and the spectrum of symptoms ranges widely, from dyspnea, sequelae of lung inflammation due to severe acute respiratory syndrome coronavirus 2 infection to “brain fog” and cognitive impairment due to chronic damage of the central nervous system [2]. The symptoms of long COVID are diverse, and the mechanisms involved remain unclear, making it difficult to establish a treatment strategy for these patients [2]. Many countries have adopted complementary and alternative medicine (CAM) in their healthcare services during the COVID-19 pandemic [3–5], and surveys from different regions have reported a significant number of individuals using CAM interventions as a means to boost immunity and prevent acute COVID-19 infection [6, 7]. CAM interventions are expected to play a significant role in the management of long COVID. Therefore, understanding the efficacy and safety of CAM interventions is urgent and necessary for their implementation.

The present study comprised a scoping review and bibliometric analysis to explore the status of research evidence for the use of CAM interventions for long COVID. The study objectives were to identify publications and evidence for various CAM interventions for the treatment of long COVID and long-term management of survivors of COVID-19 infection. Furthermore, the current research status of long COVID was overviewed using bibliometric analysis.

## 2. Materials and Methods

### 2.1. Search and Selection of Published Articles

Core medical databases, including MEDLINE, Embase, and Cochrane Library, were searched from their inception until November 2021 to identify publications related to long COVID and long-term management using CAM interventions for COVID-19 survivors. The following PICO components were used for the literature searches:

Population: patients with long COVID symptoms, such as fatigue, mild cognitive dysfunction, anosmia, dysgeusia, and other pulmonary dysfunction or neurological or psychological problems after recovery from acute COVID-19 infection.

Intervention: any type of CAM intervention, including traditional medicine, mind-body therapy, homeopathy, and dietary supplements. Limitations were not imposed on the type of comparators and outcomes of the included publications.

Publication type: any type of publication, including systematic reviews (SRs), randomized controlled trials (RCTs), and observational studies, as well as study protocols were included for human studies.

The search strategy for each database was developed according to the specific features of each database using the keywords “CAM interventions,” “long COVID,” and “related symptoms” (Supplementary File 1). The titles and abstracts of the publications were independently reviewed by two authors (S-RK and T-HK), and hard copies of potential articles were assessed for selection. Decisions about selection of the publications were made in the following discussion.

### 2.2. Data Extraction

In the present scoping review, data of publications, including condition, type of publication, type of the study, number of patients, country of the authors, intervention types, and description of interventions (frequency, duration, potential effectiveness, and safety) were extracted from the included publications. To conduct a more productive analysis, we searched the Web of Science (WoS) database for each study which was located through MEDLINE, Embase, and Cochrane Library database searching. Bibliometric analyses were conducted by retrieving citation information and references of the included articles in the WoS database format. Citation information included keywords plus, which is a specific index term for each publication defined by the WoS [8]. Two authors (S-RK and T-HK) independently extracted data, and the third author (SOK) made a final decision about instances of disagreement.

### 2.3. Bibliometric Analysis

Bibliometric analyses were conducted by narratively analyzing information about the included publications. Relevant sources (journals), top 20 authors, relevant institutions, country of the corresponding author, country contribution, keywords, and title words were narratively analyzed. The current research trend was identified using a three-field plot, which links the title, authors, and country of the corresponding author in all included publications. Word cloud, which shows the most relevant keywords of the publication, was also used. Trends in publications by year of publication were not analyzed because the COVID-19 pandemic occurred after 2019. The degree of collaboration, which can be calculated as the ratio of multiauthored articles to the sum of single and multiauthored articles, was suggested for identifying how active research collaboration was during the last two years. To access the pattern of co-authorship, we calculated the co-authorship index (CAI). In addition to this, the collaborative coefficient (CC) was calculated for assessing dominant patterns of single-authored or multiauthored publications [9]. We analyzed publication data using the Bibliometrix package for *R* (version 3.1).

## 3. Results

### 3.1. Summary of the Included Publications

**Figure 1:**
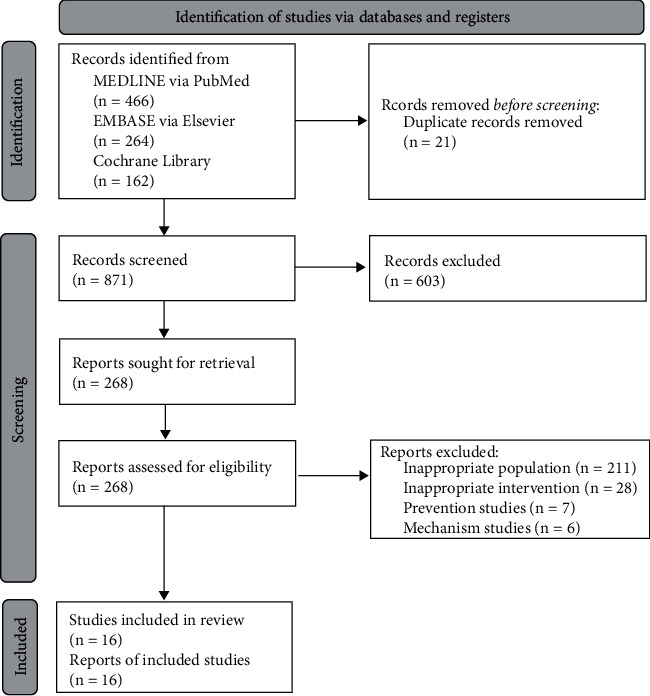
Study flow diagram.

The electronic database searches identified 16 publications, including two clinical studies [10, 11] and 14 study protocols of SRs or clinical studies (Figure 1) [12–25]. Among the 13 published protocols, most were protocols for SRs and meta-analyses and only two were RCT protocols [15, 23]. Dyspnea or pulmonary dysfunction were the target conditions of the protocols in five publications [12, 15, 20, 23, 24], and the quality of life assessment was present in six publications [13, 16, 18, 21, 22, 25]. Olfactory dysfunction [17] or psychological symptoms [14] were assessed in one study each. Various symptoms were targeted in one publication (Table 1) [26]. Among the two clinical studies, one was a prospective case-control study [10], and the other was a single case report [11]; both assessed the clinical effects of Chinese herbal medicine (CHM) decoctions for lung inflammation. In these two studies, CHM was reported to be effective in relieving symptoms of pulmonary dysfunction in patients after COVID-19 infection [10, 11].

### 3.2. Bibliometric Analysis

The 16 publications included in the analysis were published between 2020 and 2021, with an average of 0.438 years from publication as of November 2021. The average number of citations per document was 1.125. There were 33 extracted keywords plus and 42 authors' keywords. A total of 101 authors were included in all the publications, with an average of 6.31 authors per publication (Supplementary File 2). Evaluation of publication sources revealed “Medicine” as the journal most frequently published in (*n* = 12), followed by “Chinese Journal of Integrative Medicine” (*n* = 1), “Infectious Diseases of Poverty” (*n* = 1), “Journal of Integrative Medicine” (*n* = 1), and “Trials” (*n* = 1) (Supplementary File 3). The authors with the most publications were Chen Y (*n* = 3), Chi WX (*n* = 3), Luo ZY (*n* = 3), Wang LN (*n* = 3), Wen DP (*n* = 3), and Zhu XY (*n* = 3) (Table 2). The most common author affiliations were “Beijing University of Chinese Medicine” (*n* = 7), “Chengdu University of Traditional Chinese Medicine” (*n* = 5), “Capital Medical University” (*n* = 4), “Tianjin University of Traditional Chinese Medicine” (*n* = 4), “Beijing University” (*n* = 3), “Dongguk University” (*n* = 3), “Hospital of Chengdu University of Traditional Chinese Medicine” (*n* = 3), “Shandong University of Traditional Chinese Medicine” (*n* = 3), and “Shanghai University of Traditional Chinese Medicine” (*n* = 3) (Supplementary File 4). China was the most common author country, and most studies were published in China (*n* = 14), whereas the Republic of Korea and India had only one publication each (Supplementary File 5).

Analysis of the keywords revealed that “COVID-19” (*n* = 10) and “systematic review” (*n* = 10) were the most frequently used and “Tai Chi” (*n* = 3), “acupuncture” (*n* = 2), and “Traditional Chinese medicine” (*n* = 2) were the most frequently used keywords for the intervention (Supplementary File 6). Furthermore, “protocol” (*n* = 13), “review” (*n* = 12), “systematic” (*n* = 12), “COVID” (*n* = 11), “patients” (*n* = 10), and “meta-analysis” (*n* = (9) were the most frequently used title words (Supplementary File 7). The three-field plot indicated that the current trend of research published in this area was driven by study protocols written by Chinese, Korean, and Indian authors (Supplementary File 8). Word cloud analysis showed that protocols for long COVID symptoms and CAM interventions were positioned in the center of the keywords in the included publications (Supplementary File 9).

Between 2020 and 2021, the degree of collaboration was calculated to be 1, which means that all the published articles had multiauthors and there was no single-authored article. The CAI was 100 which implied that the pattern of co-authorship of the included literature between 2020 and 2021 was similar to that of the world average. The CC was calculated to be 0.8607, which suggested that multiauthored publications were dominant in this research area.

## 4. Discussion

The present scoping review and bibliometric analysis revealed a lack of published studies about use of CAM on long COVID and long-term management of COVID-19 survivors. Only 16 publications were available, including two observational studies that examined the effects of CHM treatment for recovery from lung inflammation after acute COVID-19 infection and 14 protocols for RCTs and SRs. In addition, the clinical effectiveness of CHM decoctions for lung inflammation was found to be suggested as limited evidence. Most prevalent and severe long COVID symptoms including brain fog, fatigue, and olfactory dysfunctions were not tested or were not evaluated, which suggested that urgent evaluation would be necessary for this area. From the bibliometric analysis, we found that China was the most active country where most research literature has been published already. The most frequently published journal was “Medicine,” which may be because it publishes original research protocols. Several indicators for co-authorship suggested that multiauthored publication was dominant between 2020 and 2021. Frequently tested interventions were CHM, acupuncture, and Tai Chi. These results indicate that research on CAM interventions for long COVID by Chinese researchers has focused on TCM interventions. Furthermore, pulmonary rehabilitation and improvement of quality of life were reported to be the main target of the CAM interventions assessed in these publications. In addition to this, the current research trend in this area was mainly driven by Chinese, Korean, and Indian authors, and the main publication types were protocols of RCTs or SRs for long COVID symptoms. This suggests that more time will be required until the evidence for CAM treatments to handle these long COVID symptoms to be prepared.

The present study has some limitations. First, only a small number of publications are currently available; therefore, any evidence for the effectiveness and safety of CAM interventions for long COVID cannot be suggested in the present study. Long COVID includes a range of symptoms; however, the publications included in the present study only covered a limited number of symptoms. RCTs and SRs for various symptoms of long COVID are required in the future. Second, most of the publications used protocols that may reflect the current research status. However, we did not assess the registry for clinical trials and SRs, which contained research protocols. The primary objectives of the present study were to assess the publication status of this research area and conduct a bibliometric analysis; therefore, we did not include a search of the registries. Future studies should include clinical trials and SR registries to include more diverse studies. Third, there could be publication bias due to the limited searching strategy of this study. We only assessed core databases, and there may be more studies that have been published in local databases. These points need to be considered when interpreting the results of our study.

Long COVID is an emerging health problem, and the socioeconomic burden of this condition is expected to be severe [2]. Therefore, an appropriate therapeutic plan needs to be prepared for the post-COVID-19 era. The use of CAM interventions for various diseases and conditions is increasing worldwide; however, the evidence supporting their appropriate use remains insufficient [27]. As COVID-19 and long COVID are new health problems, there is a lack of evidence in the field of CAM. Considering that CAM interventions are expected to be used frequently for long COVID, the substantial gap between knowledge and practice should be resolved through rigorous clinical studies and RCTs.

In conclusion, there is a lack of published studies about the effectiveness and safety of CAM interventions for long COVID. Original studies on CAM interventions, including RCTs and SRs, are required in the future to provide evidence for their use in this condition.

## Figures and Tables

**Table 1:** Summary of the included studies.

Study ID	Target condition or outcomes	Type of the study (review, RCT, observational study, case report)	Number of patients	Country	Intervention type	Frequency and duration of the treatment	Summary of the effectiveness (quote)	Summary of the safety (quote)
*Clinical studies*								
Li et al. [10]	Lung inflammation after COVID-19 infection	Prospective case-control study	96 (CM: 64/control: 32)	China	CHM decoction	CHM decoction 150 ml twice a day for 28 days	“Patients with COVID-19 in convalescence had symptoms and lung inflammation after hospital discharge and recovered with time prolonging. CM could improve lung inflammation for early recovery.”	NR
Zhi et al. [11]	Lung inflammation and pulmonary fibrosis after COVID-19 infection	Case report	1 case	China	CHM decoction	CHM decoction 150 ml three times a day for one month	“After one-month of oral treatment with traditional Chinese medicine decoction, without using other drugs, the lung inflammatory exudate, pulmonary fibrosis, and the quality of life of a 61-year-old female patient with coronavirus disease 2019 (COVID-19) were significantly improved.”	No adverse events occurred
*Study protocols*								
Chi et al. [12]	Dyspnea after ventilator weaning in the patients recovering from COVID-19 infection	Protocol for the systematic review and meta-analysis	NA	China	Acupuncture	NR	NA	NA
Ding et al. [13]	Quality of life in the patients recovering from COVID-19 infection	Protocol for the systematic review and meta-analysis	NA	China	Dance-based mind-motor activities including Tai chi, Baduanjin, Qigong, Yijinjing, Daoyin, and Tango dance program	NR	NA	NA
Kim et al. [14]	Psychological sequelae in the patients recovering from COVID-19 infection	Protocol for systematic review and meta-analysis	NA	Republic of Korea	THM	NR	NA	NA
Lu et al. [15]	Pulmonary fibrosis in the patients recovering from COVID-19 infection	Protocol for RCT	514 (TCM: 257/Placebo: 257)	China	TCM herbal granule	TCM herbal granule three times a day for 12 months	NA	NA
Luo et al. [16]	Quality of life in the elderly patients recovering from COVID-19 infection	Protocol for the systematic review and meta-analysis	NA	China	Tai Chi such as Yang's Tai Chi, Chen's Tai Chi and other types of Tai Chi	NR	NA	NA
Ma et al. [17]	Olfactory dysfunction induced by viral infection	Protocol for the systematic review and meta-analysis	NA	China	TCM interventions including TCM decoction, acupuncture, moxibustion, massage and cupping	NR	NA	NA
Ma et al. [18]	Quality of life in the patients recovering from COVID-19 infection	Protocol for the systematic review and meta-analysis	NA	China	Baduanjin exercise	NR	NA	NA
Shi et al. [19]	Various symptoms in the patients recovering from COVID-19 infection	Protocol for the systematic review and meta-analysis	NA	China	Tai Chi	NR	NA	NA
Sun et al. [20]	Lung ventilation function in the patients recovering from COVID-19 infection	Protocol for the systematic review and meta-analysis	NA	China	CHM	NR	NA	NA
Wang et al. [21]	Quality of life in the patients recovering from COVID-19 infection	Protocol for the systematic review and meta-analysis	NA	China	Meditative movements such as Tai Chi or qigong or Tai Chi combined with qigong or yoga	NR	NA	NA
Wen et al. [25]	Quality of life in the patients recovering from COVID-19 infection	Protocol for the systematic review and meta-analysis	NA	China	Acupuncture	NR	NA	NA
Wu et al. [22]	Quality of life in the patients recovering from COVID-19 infection	Protocol for the systematic review and meta-analysis	NA	China	Massage including tuina and manipulation	NR	NA	NA
Yadav et al. [23]	Pulmonary function in the patients recovering from COVID-19 infection	Protocol for RCT	110 (55/55)	India	Ayurveda and yoga	Ayurveda interventions including Agastya Haritaki 6 g and Ashwagandha tablet 500 mg twice daily and two sessions of yoga (morning 30 minutes and evening 15 minutes) daily for 90 days	NA	NA
Zhu et al. [24]	Pulmonary function in the patients recovering from COVID-19 infection	Protocol for the systematic review and meta-analysis	NA	China	Tai Chi such as Yang's Tai Chi, Chen's Tai Chi and other types of Tai Chi	NR	NA	NA

CHM: Chinese herbal medicine; NA: not applicable; NR: not reported; RCT: randomized controlled trial; TCM: traditional Chinese medicine; THM: traditional herbal medicine.

**Table 2:** Top 20 relevant authors.

Authors	Articles
CHEN Y	3
CHI WX	3
LUO ZY	3
WANG LN	3
WEN DP	3
ZHU XY	3
DONG YT	2
HUANG J	2
LI HY	2
LI L	2
WU L	2
ZHANG J	2
ZHANG Y	2
CHEN H	1
CHEN HJ	1
CHEN XC	1
CHENG PY	1
CHENG XX	1
CHOI J	1
DAI SX	1

## Data Availability

All data of this study are included in the appendix file.

## Supplementary Materials

Supplementary Materials
*Supplementary file 1.* Search strategies used in each database. *Supplementary file 2.* The main information of the included publications. *Supplementary file 3.* Most relevant sources (journals). *Supplementary file 4.* Most relevant affiliations of the authors. *Supplementary file 5.* Country scientific contribution. *Supplementary file 6.* Authors' keywords which were used in more than two articles. *Supplementary file 7.* Words of titles which were used in more than two articles. *Supplementary file 8.* Three field plots (title-author-country). *Supplementary file 9.* Word cloud.
