# Supplementary material for: Complementary and Alternative Medicine for Long COVID: Scoping Review and Bibliometric Analysis
Source: Evid Based Complement Alternat Med. 2022 Aug 4;2022:7303393. doi: 10.1155/2022/7303393 (PMC9371860; doi:10.1155/2022/7303393)

**Supplementary file 1. Search strategies used in each database**

**MEDLINE via PubMed (19/Nov/2021)**

|  | Searches | Results |
| --- | --- | --- |
| #1 | ((("brain"[MeSH Terms] OR "brain"[All Fields] OR "brains"[All Fields] OR "brain s"[All Fields]) AND "fog"[All Fields]) OR ("Post"[All Fields] AND "COVID"[All Fields] AND "19"[All Fields]) OR 7[UID] OR ("Long"[All Fields] AND ("sars cov 2"[MeSH Terms] OR "sars cov 2"[All Fields] OR "COVID"[All Fields] OR "covid 19"[MeSH Terms] OR "covid 19"[All Fields])) OR ("Post"[All Fields] AND ("virally"[All Fields] OR "virals"[All Fields] OR "virology"[MeSH Terms] OR "virology"[All Fields] OR "viral"[All Fields]) AND ("syndrom"[All Fields] OR "syndromal"[All Fields] OR "syndromally"[All Fields] OR "syndrome"[MeSH Terms] OR "syndrome"[All Fields] OR "syndromes"[All Fields] OR "syndrome s"[All Fields] OR "syndromic"[All Fields] OR "syndroms"[All Fields])) OR ("Long"[All Fields] AND ("sars cov 2"[MeSH Terms] OR "sars cov 2"[All Fields] OR "COVID"[All Fields] OR "covid 19"[MeSH Terms] OR "covid 19"[All Fields])) OR ("Post"[All Fields] AND ("sars cov 2"[MeSH Terms] OR "sars cov 2"[All Fields] OR "COVID"[All Fields] OR "covid 19"[MeSH Terms] OR "covid 19"[All Fields])) OR ("ongoing"[All Fields] AND ("symptomatic"[All Fields] OR "symptomatically"[All Fields] OR "symptomatics"[All Fields]) AND ("covid 19"[All Fields] OR "covid 19"[MeSH Terms] OR "covid 19 vaccines"[All Fields] OR "covid 19 vaccines"[MeSH Terms] OR "covid 19 serotherapy"[All Fields] OR "covid 19 serotherapy"[Supplementary Concept] OR "covid 19 nucleic acid testing"[All Fields] OR "covid 19 nucleic acid testing"[MeSH Terms] OR "covid 19 serological testing"[All Fields] OR "covid 19 serological testing"[MeSH Terms] OR "covid 19 testing"[All Fields] OR "covid 19 testing"[MeSH Terms] OR "sars cov 2"[All Fields] OR "sars cov 2"[MeSH Terms] OR "severe respiratory syndrome coronavirus 2"[All Fields] OR "ncov"[All Fields] OR "2019 ncov"[All Fields] OR (("coronavirus"[MeSH Terms] OR "coronavirus"[All Fields] OR "cov"[All Fields]) AND 2019/11/01:3000/12/31[Date - Publication]))) OR (("chronic"[All Fields] OR "chronical"[All Fields] OR "chronically"[All Fields] OR "chronicities"[All Fields] OR "chronicity"[All Fields] OR "chronicization"[All Fields] OR "chronics"[All Fields]) AND ("sars cov 2"[MeSH Terms] OR "sars cov 2"[All Fields] OR "COVID"[All Fields] OR "covid 19"[MeSH Terms] OR "covid 19"[All Fields])) OR ("Long"[All Fields] AND "haul"[All Fields] AND ("sars cov 2"[MeSH Terms] OR "sars cov 2"[All Fields] OR "COVID"[All Fields] OR "covid 19"[MeSH Terms] OR "covid 19"[All Fields])) OR (("complications"[MeSH Subheading] OR "complications"[All Fields] OR "sequelae"[All Fields] OR "sequela"[All Fields] OR "sequelaes"[All Fields] OR "sequelas"[All Fields]) AND ("sars cov 2"[MeSH Terms] OR "sars cov 2"[All Fields] OR "COVID"[All Fields] OR "covid 19"[MeSH Terms] OR "covid 19"[All Fields])) OR (("complications"[MeSH Subheading] OR "complications"[All Fields] OR "sequelae"[All Fields] OR "sequela"[All Fields] OR "sequelaes"[All Fields] OR "sequelas"[All Fields]) AND ("sars cov 2"[MeSH Terms] OR "sars cov 2"[All Fields] OR "sars cov 2"[All Fields])) OR (("sars cov 2"[MeSH Terms] OR "sars cov 2"[All Fields] OR "COVID"[All Fields] OR "covid 19"[MeSH Terms] OR "covid 19"[All Fields]) AND ("survivor s"[All Fields] OR "survivors"[MeSH Terms] OR "survivors"[All Fields] OR "survivor"[All Fields]))) AND ("Complementary Therapies"[MeSH Terms] OR "CAM"[All Fields] OR ("Dietary Supplements"[MeSH Terms] OR ("plants, medicinal"[MeSH Terms] OR "Herbals as Topic"[MeSH Terms]) OR "Probiotics"[MeSH Terms] OR "Vitamins"[MeSH Terms] OR "Minerals"[MeSH Terms]) OR ("Meditation"[MeSH Terms] OR "Hypnosis"[MeSH Terms] OR "Relaxation Therapy"[MeSH Terms] OR "Qigong"[MeSH Terms] OR "Exercise Movement Techniques"[MeSH Terms] OR "Musculoskeletal Manipulations"[MeSH Terms]) OR ("Acupuncture"[MeSH Terms] OR "Acupuncture Therapy"[MeSH Terms] OR "acupuncture, ear"[MeSH Terms] OR "Acupuncture Points"[MeSH Terms] OR "Massage"[MeSH Terms] OR "manipulation, spinal"[MeSH Terms]) OR ("Yoga"[MeSH Terms] OR "Tai Ji"[MeSH Terms] OR "Dance Therapy"[MeSH Terms] OR "Art Therapy"[MeSH Terms]) OR ("Chiropractic"[MeSH Terms] OR "manipulation, chiropractic"[MeSH Terms] OR "manipulation, osteopathic"[MeSH Terms]) OR ("medicine, east asian traditional"[MeSH Terms] OR "medicine, korean traditional"[MeSH Terms] OR "medicine, tibetan traditional"[MeSH Terms] OR "medicine, mongolian traditional"[MeSH Terms] OR "medicine, african traditional"[MeSH Terms] OR "medicine, chinese traditional"[MeSH Terms] OR "medicine, traditional"[MeSH Terms] OR "medicine, ayurvedic"[MeSH Terms] OR "Homeopathy"[MeSH Terms] OR "Naturopathy"[MeSH Terms]) OR ("int j complement altern med"[Journal] OR "evid based complement alternat med"[Journal] OR "bmc complement altern med"[Journal] OR ("complementary"[All Fields] AND "and"[All Fields] AND "alternative"[All Fields] AND "medicine"[All Fields]) OR "complementary and alternative medicine"[All Fields])) | 466 |

**EMBASE via Elsevier (19/Nov/2021)**

|  | Searches | Results |
| --- | --- | --- |
| #1 | ‘coronavirus disease 2019'/exp OR (corona AND virus AND 19) OR covid OR (long AND covid) OR (brain AND fog) | 202427 |
| #2 | 'complementary and alternative medicine'/exp OR 'acupuncture'/exp OR 'dietary supplement'/exp OR 'probiotic agent'/exp OR 'meditation'/exp OR 'hypnosis'/exp OR (('music'/exp OR music) AND ('therapy'/exp OR therapy)) OR 'relaxation training'/exp OR 'pilates'/exp OR 'massage'/exp OR 'spine manipulation'/exp OR 'yoga'/exp OR 'tai chi'/exp OR 'dance therapy'/exp OR 'chiropractic'/exp OR 'traditional medicine'/exp OR 'ayurveda'/exp OR 'homeopathy'/exp OR 'naturopathy'/exp | 293753 |
| #3 | ‘systematic review’ | 405789 |
| #4 | #1 AND #2 AND #3 | 264 |

**Cochrane library (19/Nov/2021)**

|  | Searches | Results |
| --- | --- | --- |
| #1 | COVID-19 | 8042 |
| #2 | long COVID-19 syndrome | 223 |
| #3 | COVID-19 stress syndrome | 92 |
| #4 | COVID-19 post-intensive care syndrome | 11 |
| #5 | sars cov | 599 |
| #6 | COVID | 8163 |
| #7 | corona | 876 |
| #8 | #1 OR #2 OR #3 OR #4 OR #5 OR #6 OR #7 | 8676 |
| #9 | Complementary Therapies | 3876 |
| #10 | CAM | 2860 |
| #11 | Dietary Supplements | 14828 |
| #12 | plants, medicinal | 1625 |
| #13 | Probiotics | 6018 |
| #14 | Vitamins | 7841 |
| #15 | Minerals | 2343 |
| #16 | Meditation | 3444 |
| #17 | Hypnosis | 1898 |
| #18 | Relaxation Therapy | 7070 |
| #19 | Qigong | 609 |
| #20 | Exercise Movement Techniques | 1130 |
| #21 | Musculoskeletal Manipulations | 616 |
| #22 | Acupuncture | 17798 |
| #23 | Massage | 6227 |
| #24 | Manipulation, spinal | 1371 |
| #25 | Yoga | 4154 |
| #26 | Tai Ji | 484 |
| #27 | Dance Therapy | 542 |
| #28 | Art Therapy | 9973 |
| #29 | Chiropractic | 1388 |
| #30 | Osteopathic | 1000 |
| #31 | Traditional medicine | 28161 |
| #32 | Ayurveda | 873 |
| #33 | Homeopathy | 931 |
| #34 | Naturopathy | 168 |
| #35 | Complementary and alternative medicine | 5479 |
| #36 | #9 OR #10 OR #11 OR #12 OR # 13 OR #14 OR #15 OR #16 OR #17 OR #18 OR #19 OR #20 OR #21 OR #22 OR #23 OR #24 OR #25 OR #26 OR #27 OR #28 OR #29 OR #30 OR #31 OR #32 OR #33 OR #34 OR #35 | 333798 |
| #37 | #8 AND #36 in Trials | 1627 |
| #38 | Cochrane Reviews | 162 |

**Supplementary file 2. Main information of the included publications**

| **Description** | **Results** |
| --- | --- |
| Timespan | 2020:2021 |
| Documents | 16 |
| Average years from publication | 0.438 |
| Average citations per documents | 1.125 |
| Average citations per year per doc | 0.7812 |
| References | 352 |
| **DOCUMENT CONTENTS** | |
| Keywords Plus (ID) | 33 |
| Author's Keywords (DE) | 42 |
| **AUTHORS** |  |
| Authors | 101 |
| Author Appearances | 120 |
| Authors of single-authored documents | 0 |
| Authors of multi-authored documents | 101 |
| **AUTHORS COLLABORATION** | |
| Single-authored documents | 0 |
| **Documents per Author** | 0.158 |
| Authors per Document | 6.31 |
| Co-Authors per Documents | 7.5 |

**Supplementary file 3. Most relevant sources (journals)**

|  | |
| --- | --- |
| **Sources** | **Articles (n)** |
| Medicine | 12 |
| Chinese journal of integrative medicine | 1 |
| Infectious diseases of poverty | 1 |
| Journal of integrative medicine | 1 |
| Trials | 1 |

**Supplementary file 4. Most relevant affiliations of the authors**

| **Affiliations** | **Articles** |
| --- | --- |
| Beijing university of Chinese medicine | 7 |
| Chengdu university of Chinese medicine | 5 |
| Capital medical university | 4 |
| Tianjin university of traditional Chinese medicine | 4 |
| Beijing sport university | 3 |
| Dongguk university | 3 |
| Hospital of Chengdu university of traditional Chinese medicine | 3 |
| Shandong university of traditional Chinese medicine | 3 |
| Shanghai university of traditional Chinese medicine | 3 |
| Gachon university | 2 |
| Guizhou university of traditional Chinese medicine | 2 |
| Chongqing Beibei district traditional Chinese medicine hospital | 1 |
| Central council for research in Ayurvedic sciences | 1 |
| Characteristic medical center of Chinese people's armed police force | 1 |
| Chengdu medical college | 1 |
| China academy of Chinese medical sciences | 1 |
| Guangzhou women and children’s medical center | 1 |
| Hebei north university | 1 |
| Ilsan medical center | 1 |
| Jinan university | 1 |

**Supplementary file 5. Country scientific contribution**


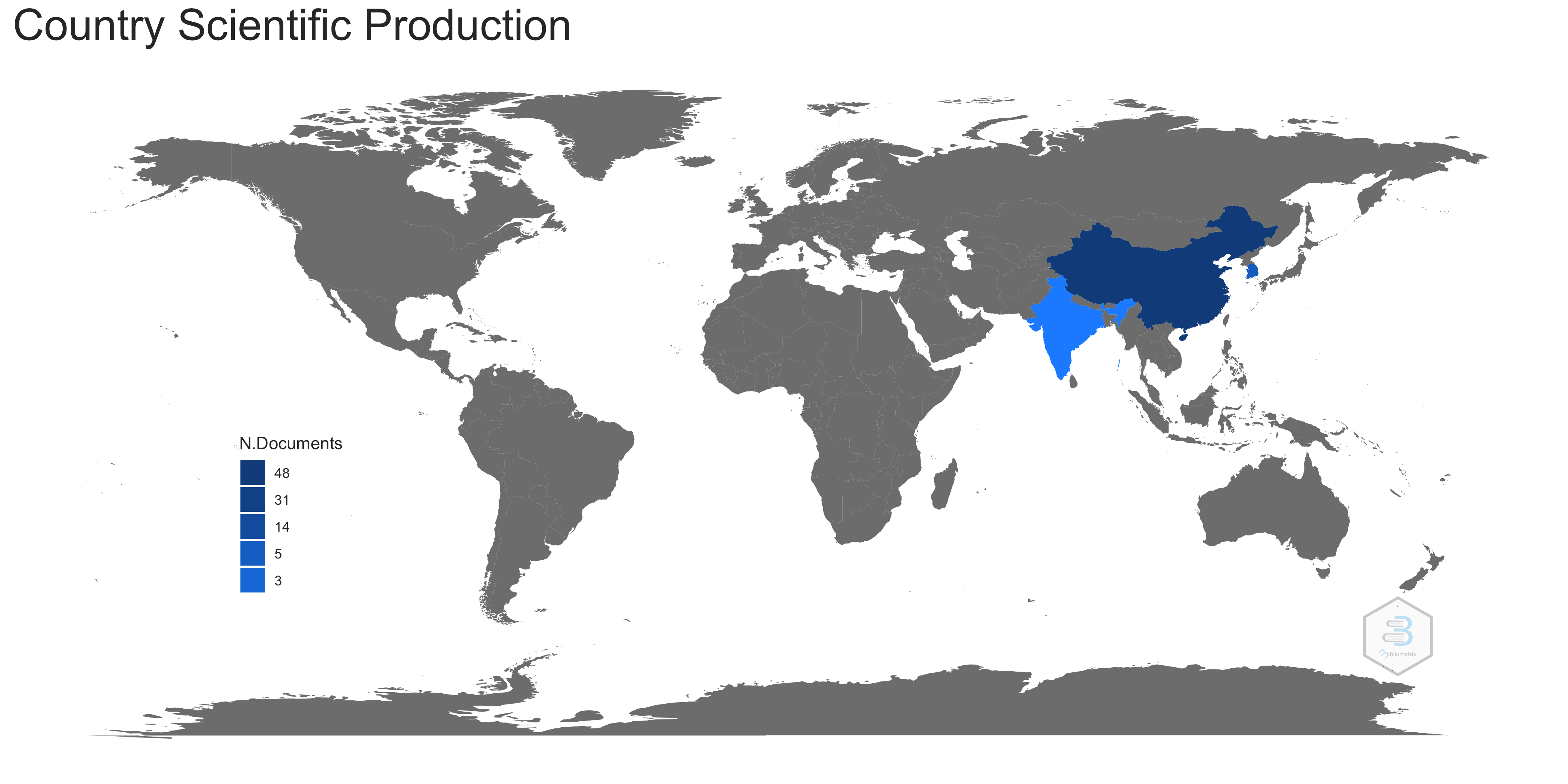


**Supplementary file 6. Author’s keywords which were used in more than two articles**

| **Words** | **Occurrences** |
| --- | --- |
| COVID-19 | 10 |
| Systematic review | 10 |
| Meta-analysis | 5 |
| Coronavirus disease 2019 | 4 |
| Protocol | 3 |
| Tai chi | 3 |
| Acupuncture | 2 |
| Corona virus disease 2019 | 2 |
| Pulmonary fibrosis | 2 |
| Traditional Chinese medicine | 2 |

**Supplementary file 7. Words of titles which were used in more than two articles**

| **Words** | **Occurrences** |
| --- | --- |
| Protocol | 13 |
| Review | 12 |
| Systematic | 12 |
| COVID | 11 |
| Patient | 11 |
| Meta-analysis | 9 |
| Effect | 8 |
| Medicine | 7 |
| Life | 6 |
| Quality | 6 |
| Recovering | 6 |
| Chinese | 5 |
| Disease | 4 |
| Traditional | 4 |
| Tai-Chi | 3 |
| Convalescent | 3 |
| Efficacy | 3 |
| Acupuncture | 2 |
| Corona | 2 |
| Coronavirus | 2 |
| Elderly | 2 |
| Fibrosis | 2 |
| Herbal | 2 |
| Period | 2 |
| Pulmonary | 2 |
| Randomized | 2 |
| Recovery | 2 |
| Sequelae | 2 |
| Study | 2 |
| Treatment | 2 |
| Trial | 2 |
| Virus | 2 |

**Supplementary file 8. Three field plot (title-author-country)**


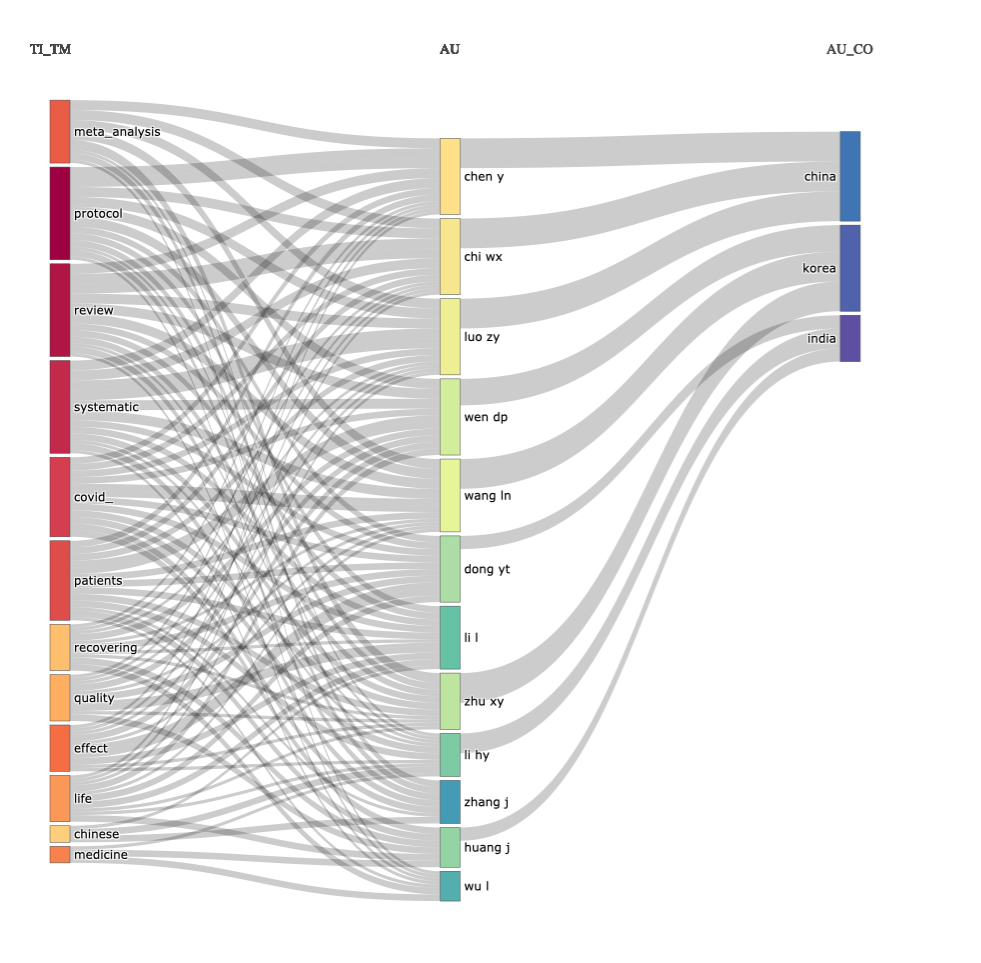


**Supplementary file 9. Wordcloud**


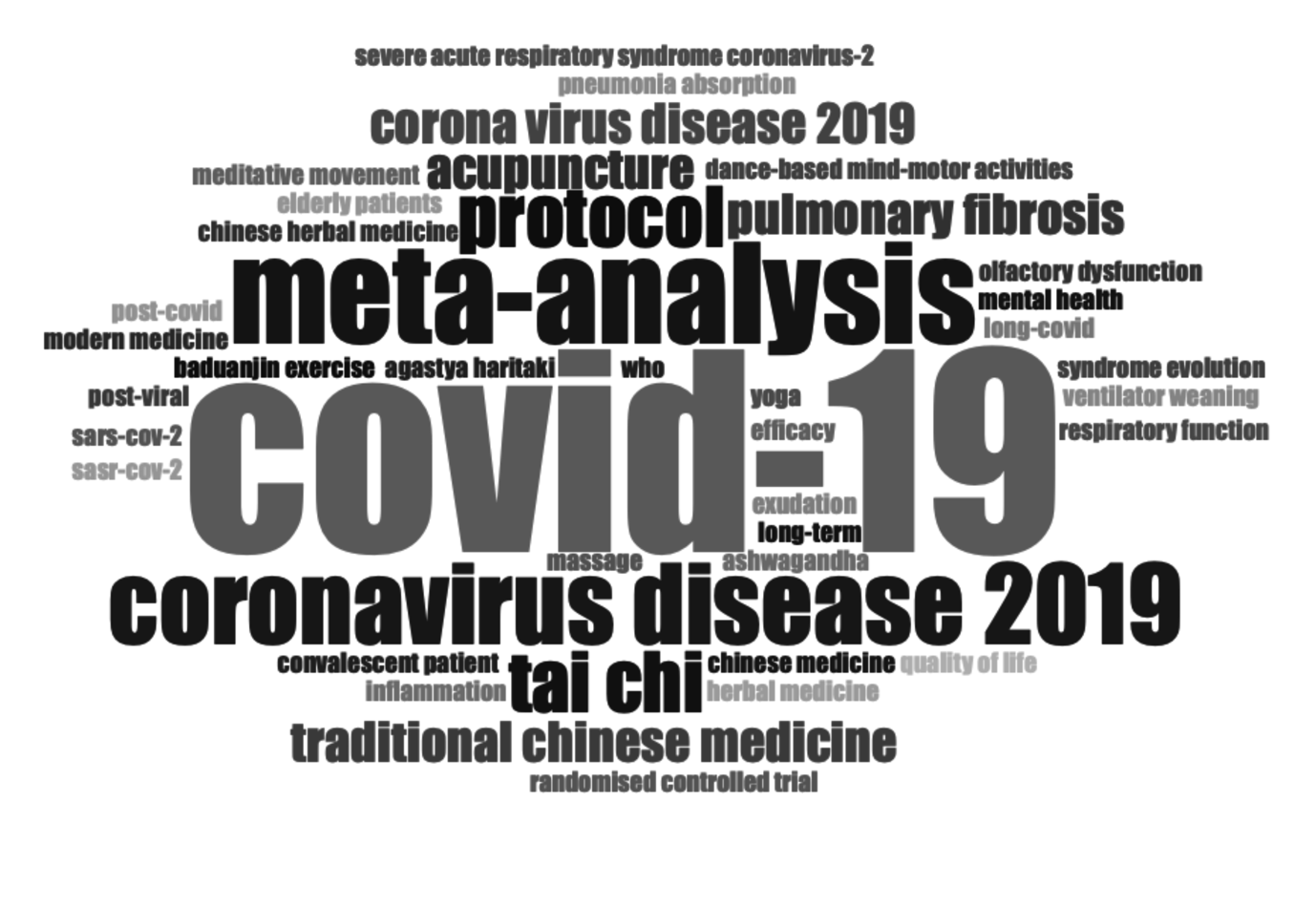

Supplement: Supplementary Materials — Supplementary file 1. Search strategies used in each database. Supplementary file 2. The main information of the included publications. Supplementary file 3. Most relevant sources (journals). Supplementary file 4. Most relevant affiliations of the authors. Supplementary file 5. Country scientific contribution. Supplementary file 6. Authors' keywords which were used in more than two articles. Supplementary file 7. Words of titles which were used in more than two articles. Supplementary file 8. Three field plots (title-author-country). Supplementary file 9. Word cloud. [file 7303393.f1.docx]
